# Hepatitis E Virus Immunopathogenesis

**DOI:** 10.3390/pathogens10091180

**Published:** 2021-09-13

**Authors:** Kush Kumar Yadav, Scott P. Kenney

**Affiliations:** Center for Food Animal Health, Department of Animal Sciences, The Ohio State University, Wooster, OH 44691, USA; yadav.94@osu.edu

**Keywords:** hepatitis E, pregnant, immunocompromised, in vitro, in vivo, models

## Abstract

Hepatitis E virus is an important emerging pathogen producing a lethal impact on the pregnant population and immunocompromised patients. Starting in 1983, it has been described as the cause for acute hepatitis transmitted via the fecal–oral route. However, zoonotic and blood transfusion transmission of HEV have been reported in the past few decades, leading to the detailed research of HEV pathogenesis. The reason behind HEV being highly virulent to the pregnant population particularly during the third trimester, leading to maternal and fetal death, remains unknown. Various host factors (immunological, nutritional, hormonal) and viral factors have been studied to define the key determinants assisting HEV to be virulent in pregnant and immunocompromised patients. Similarly, chronic hepatitis is seen particularly in solid organ transplant patients, resulting in fatal conditions. This review describes recent advances in the immunopathophysiology of HEV infections in general, pregnant, and immunocompromised populations, and further elucidates the in vitro and in vivo models utilized to understand HEV pathogenesis.

## 1. Introduction

**Figure 1:**
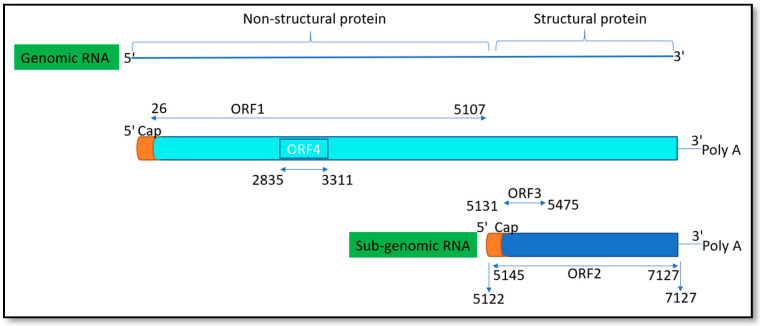
Schematic diagram of HEV genome genotype 1. HEV genome is comprised of 5′ cap and 3′ poly A tail. ORF1 consists of nucleotide (nt) 26–5107 and encodes a polyprotein of 1694 amino acids (aa) in length, which encodes several putative protein domains. ORF2 (nt 5145–7127) and ORF3 (nt 5131–5475) are translated from the sub-genomic RNA. ORF4 overlaps ORF1 in a different reading frame and is only produced during endoplasmic reticulum stress.

The hepatitis E virus (HEV) is a positive-sense single-stranded RNA virus, ~7.2 kb in length. It is a member of the genus hepevirus of the family *Hepeviridae* [1]. Computer-based genome annotation initially revealed three overlapping open reading frames (ORFs), ORF1, ORF2, and ORF3; some gt1 strains additionally contain an ORF4 [2,3,4]. ORF1 encodes for nonstructural proteins and is the largest ORF with 5079 bases. ORF2 and ORF3 are translated from a sub genomic RNA and encode for structural proteins consisting of 1980 and ~342 nucleotides (Figure 1). Computer-assisted alignment of the domains in ORF1 demonstrated that there are distinct functional domains: (a) methyl transferase (MT), (b) Y domain, (c) papain-like cysteine protease (PCP), (d) proline-rich hinge domain, (e) X domain, (f) RNA helicase, and (g) RNA-dependent RNA polymerase (RdRp) [3]. Furthermore, many of their functional activities have been biochemically demonstrated [4].

Understanding the pathogenesis of HEV has been a difficult task for decades due to a lack of robust cell culture models and animal models failing to recapitulate the full disease pathology seen in humans. Although the primary route of HEV transmission is the fecal–oral route, it was unclear how the virus particles travel from gastrointestinal tract to the liver. Recently, primary cultures of intestinal cells have been shown to support HEV gt1 and HEV gt3 replication, while HEV RNA and ORF2 antigens have been detected in the intestinal crypts of a chronically infected patient [5]. These findings suggest the initial site of HEV replication to be the intestinal tract before HEV invades hepatocytes, producing hepatitis. It has been shown that most HEV particles are released at the apical membrane (bile side) [6]. Bile salts remove the lipid envelope from the virus, which is then shed naked in the stool [7]. The liver damage induced by an HEV infection may be attributed to immune mediated cytotoxic T cells and natural killer cells [8]. Furthermore, recent data also indicate that non virus-specific CD8+ T cells can be involved in liver damage [9]. Below, we have described the HEV life cycle and generalized innate immune response against positive-strand RNA viruses, so the reader can understand the likely specific innate immune response against HEV. Furthermore, pathogenesis of HEV in general, pregnant, and immunocompromised populations have been discussed while listing in vitro models and in vivo models that facilitated the study of HEV.

## 2. HEV Transmission and Replication in the Host

HEV is an enteric virus mainly transmitted through the fecal–oral route. However, blood transfusion and vertical transmission routes have been elaborated with HEV [10]. Of the two morphological forms, quasi-enveloped HEV particles are seen in cultured cells and in the environment. In addition, the virions from cell culture supernatant possess lipid and ORF3 protein, which are absent in HEV particles from feces [11]. 

Naked HEV (NHEV) virions and enveloped HEV (EHEV) virions are enterically transmitted and possibly enter the bloodstream after the first round of replication. To date, definitive cell entry receptors for HEV have not been defined [12]. In brief, heparan sulfate proteoglycan (HSPG) is utilized by NHEV, although EHEV attachment is independent of HSPG [13]. Clathrin-mediated and dynamin-2-dependent endocytosis is utilized by both morphological forms of virions for entry [13]. 

The exact mechanism of uncoating is not well understood. However, uncoating is followed by the release of the nucleic acid into the cytosol where it serves as the template for the translation of ORF1. Translation is cap-dependent, which requires the recruitment of the 40S ribosomal subunit by 7-methylguanosine cap structure at the 5′ UTR of the HEV genome. RdRp will initiate transcription of the viral genomic RNA by binding to its 3′ UTR to produce the negative sense intermediate RNA [14]. This intermediate RNA serves as the template for the synthesis of progeny positive-sense viral genomes. It has been demonstrated that Golgi-specific brefeldin A-resistant guanine nucleotide exchange factor 1 (GBF1) is required for the activity of HEV replication complexes. However, GBF1 does not colocalize with ORF1 protein, and its subcellular distribution is unmodified upon infection or overexpression of viral proteins, indicating that GBF1 is likely not recruited to replication sites [15,16]. The ubiquitin-proteasome system is known to contribute to the HEV replication, as demonstrated by replication inhibition when the system is blocked [16]. Viral encapsidation and assembly starts with interaction between capsid protein and 76nt region in the 5′ end of the genome [17]. 

**Figure 2:**
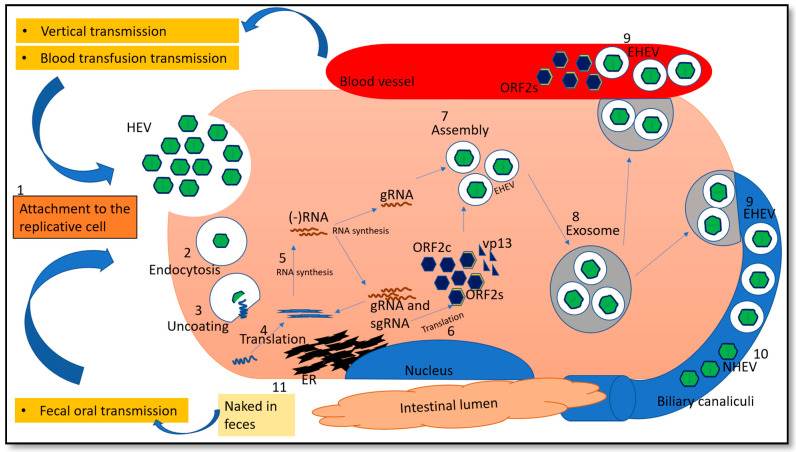
Transmission and life cycle of HEV. HEV binds cellular receptors which are still incompletely characterized. Entry is mediated by endocytosis. Uncoating is followed by the release of viral genomic RNA which serves as mRNA for ORF1 translation. RdRp synthesizes negative strand RNA (- RNA), followed by synthesis of genomic RNA (gRNA) and sub-genomic RNA (sgRNA). Translation of structural proteins occurs followed by assembly and egress. The released HEV is enveloped (EHEV), however when released into biliary canaliculi, envelope becomes degraded, and naked (NHEV) virions are released into intestines and excreted in feces. The EHEV is also released into the blood vessels. Furthermore, ORF2s (secreted form) is glycosylated and secreted into the blood stream. When such blood is transfused to naive patients or if the woman is pregnant, HEV transmission happens, which is referred as blood-borne transfusion or vertical transmission, respectively. Number 1 to 11 represents the step-by-step process occurring in the life cycle. ER—Endoplasmic reticulum.

The assembly of the viral particles (genome, capsid, ORF3) occurs, which are transported by the multivesicular bodies and released by the cellular exosomal pathway [18]. Infectious HEV particles in the form of EHEV are released from the apical side of the hepatocytes into the biliary canaliculi, where the EHEV are converted to NHEV by the bile enzymes (Figure 2). Furthermore, EHEV particles are also released into the blood via the basolateral side of the hepatocytes. Hence, NHEV can be detected only in bile and feces, but EHEV can be found in blood and urine [19]. EHEV are derived from the intracellular membrane, as suggested by the presence of trans-Golgi network protein 2 (TGOLN2), which renders the ability to escape the neutralization by capsid-specific monoclonal antibodies [20]. 

## 3. Generalized Innate Immune Response against Positive-Strand RNA Viruses

The antiviral state in the infected cell is determined by recognition of the viral pathogen, rapid production of interferons (IFNs), and pro-inflammatory cytokines. Host cells detect viral RNA using cytosolic RIG I-like receptors (RLRs) and membrane-bound Toll-like receptors (TLRs) [21,22]. Detection of viral RNA activates RLRs and triggers downstream signaling through the mitochondrial antiviral signaling (MAVS) adaptor, which is localized on the outer mitochondrial membrane [23]. Subsequently, MAVS recruits various adaptor molecules, such as stimulator of interferon genes (STING) and TNF receptor-associated factors, resulting in the formation of large signaling complexes [24]. Ultimately, this leads to the activation of kinase complexes IKKε/TBK1 and IKKα/IKKβ/IKKγ, resulting in the activation of interferon regulating factor 3 (IRF3), 7 (IRF7), and NF-κB. These transcription factors then translocate to the nucleus and initiate the expression of IFNs and pro-inflammatory cytokines [25].

In brief, the antiviral adaptive immune response is coordinated by IFNs. Three types of IFNs (I, II, III) are known. Type I IFNs consist of 13 subtypes of IFNα and single subtypes of IFNβ, IFNδ, IFNε, IFNκ, IFNτ, and IFNω. Type II IFN only contains one subtype of IFN-γ, and type III IFNs comprise four types (IFNλ1 through λ4). Although most cell types produce type I IFNs in response to viral infection, type II IFNs are specifically produced after antigenic stimulation of an expanding group of certain immune cells, including T cells, natural killer cells, dendritic cells, and macrophages [26,27]. Despite the presence of multiple IFN and receptor types, the Janus kinase signal transducer and activator of transcription (JAK/STAT) pathway is utilized by all IFNs to establish the expression of interferon-stimulated genes (ISGs) [28]. 

## 4. Innate Immune Response Escape by HEV

Even though robust immune responses exist in the host, HEV has developed multiple strategies to thwart or escape the defense mechanisms leading to the pathological condition. Hence, the interaction between HEV viral proteins and host innate immunity is crucial to understand the virulence properties of HEV and disease-enhancing factors of HEV. While studying experimentally infected chimpanzees, it has been shown that HEV triggers a stronger IFN response than the hepatitis A virus (HAV) and hepatitis C virus (HCV) [29,30]. However, all genotypes of HEV cannot be characterized as equals, as suggested by the analysis of rhesus macaque liver gene expression which demonstrated differing profiles depending on the genotype (gt1 or gt3) used for the infection [31]. In brief, 25% of the interferon-responsive genes were downregulated during early viremia following an HEV gt1 infection, including IRF3 and IRF7, or ISG15. In contrast, these same genes were upregulated during HEV gt3 infection [31]. Differences in host immune gene expression by genotype is likely due to differences in virus protein sequences altering virus–host signaling interaction.

**Figure 3:**
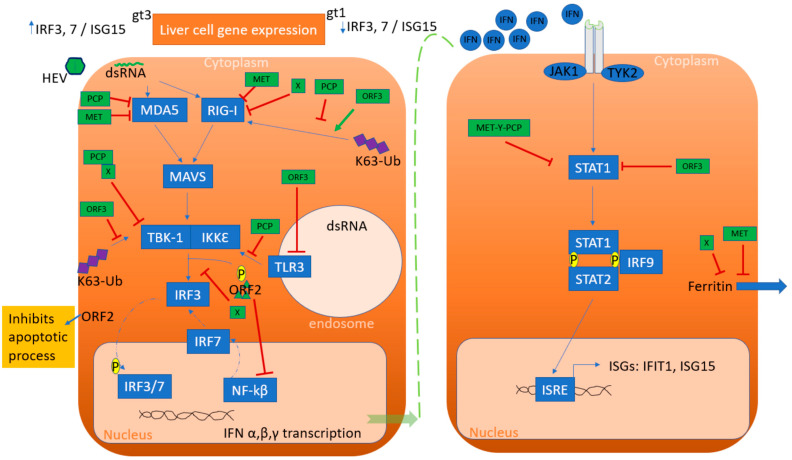
Summarization of innate immune escape by HEV. Liver cell gene expression demonstrated differentially regulated genes by genotype (gt) 1 and gt3 infection. Retinoic acid-inducible gene I (RIG-I) and melanoma differentiation-associated protein 5 (MDA5) detects the double-stranded (ds) HEV RNA, leading to type I and type III interferon (IFN) production. HEV RNA is detected by Toll-like receptor 3 (TLR3) in the endosomal compartment. Protease domain (PCP) of the ORF1 protein inhibits signaling via RIG-I and prevents IFN induction by removing ubiquitin from RIG-I and TANK binding kinase 1 (TBK-1). Methyltransferase (MET) interferes with ferritin secretion to decrease the inflammatory response and acts on RIG-I and MDA5 to reduce IFN production. X domain and capsid protein ORF2 inhibit the phosphorylation (P) of IFN regulatory protein 3 (IRF3). ORF2 has been shown to inhibit NF-kβ activity and also inhibits the apoptotic process. ORF3 stimulates the production of type I INF via RIG-I, while ORF3 interferes with TLR3 synthesis. ORF1 (MET-Y-PCP) and ORF3 both bind to STAT1 to restrict its phosphorylation and the activation of the downstream cascade, finally inhibiting ISG expression, including that of “interferon-induced protein with tetratricopeptide repeats 1 (IFIT1) and ISG15. Abbreviations: IRF3, 7 or 9: IFN regulatory protein 3, 7 or 9; IKKε (IkB-kinase-epsilon); ISRE: interferon-stimulated response element; MAVS: mitochondrial antiviral-signaling protein; STAT1 OR 2: signal transducer and activator of transcription 1 or 2; and Ub: ubiquitin.

IFN-β expression induced with poly (I:C) transfection has been demonstrated to be inhibited by HEV ORF1 in cell culture [32]. This function is credited to the papain-like cysteine protease domain (PCP) and macro domain (X) that are responsible for inactivating RIG-1 and TBK-1 as well as disrupting the phosphorylation of IRF3 [32]. Furthermore, methyltransferase and PCP have been shown to inhibit the interferon stimulation response element (ISRE) promoter activity and the expression of ISGs through inhibiting nuclear translocation and phosphorylation of STAT1 (Figure 3) [33]. Similarly, HEV-infected patients have abundant ferritin secretion in response to acute phase inflammation. However, it has been shown that the X domain is responsible for the inhibition of ferritin secretion in cell culture. Additionally, HEV replication is facilitated by RdRp (RNA-dependent RNA polymerase) and domain Y interacting with microRNA (miRNA) [34]. In brief, HEV gt1 harbors at least one microRNA target site in the RdRp region; however, HEV gt1 and gt3 does not interfere in the biogenesis of microRNA-122. However, microRNA-122 is demonstrated to facilitate the replication of HEV (gt1, gt3) in human hepatoma cells as well as non-hepatoma cells. Furthermore, inhibition of microRNA-122 molecules drastically reduced HEV (gt1, gt3) replication [34].

The structural capsid protein is encoded by ORF2, which binds the 5′ end of the genome and is involved in viral encapsidation [35]. Up to now, three forms of ORF2 have been identified, including infectious, glycosylated, and cleaved ORF2 [36]. Infectious ORF2 facilitates the entry of the virus and is known to assemble in the viral particles [37]. Furthermore, ORF2 has been shown to inhibit NF-kβ activity by inhibiting Ikβα ubiquitination [38]. Similarly, the host transmembrane protein 134 (TMEM134) interacts with ORF2 to attenuate its inhibitory effect on NF- kβ by ORF2 [39]. In addition, ORF2 can also impair the host’s apoptotic response to favor HEV infection [40]. Finally, glycosylated ORF2 and cleaved ORF2 are not associated with infectious particles and are highly stable proteins which are targeted by patient antibodies as immunological decoys [41]. 

ORF3 encodes the multifunctional phosphoprotein which acts as a viroporin known to help in cell signaling, virion morphogenesis, and egress. In vitro studies on human lung epithelial A549 cells and hepatocarcinoma Huh7 cells indicate that interferon-induced phosphorylation of STAT1 is inhibited by the ORF3 protein, blocking the synthesis of two key antiviral proteins, double-stranded (ds) RNA-activated protein kinase (PKR), and 2′,5′-oligoadenylate synthetase (2′,5′-OAS) [42,43]. The ORF3 protein enhanced type ׀ interferon production in HEK293T cells by interacting directly with the pattern recognition receptor (PRR), retinoic acid-inducible gene ׀ (RIG-׀). ORF3 of gt1 has been reported to downregulate the expression of tumor necrosis factor 1-associated death domain protein (TRADD) and receptor-interacting protein kinase 1 (RIP1), thus inhibiting TLR3-mediated activation of NF- kβ upon poly (I:C) treatment [44]. Furthermore, ORF3 inhibits expression of endogenous IFNα/β through inhibiting the expression of TLR3 and TLR7 [45]. Similarly, ORF3 inhibits the activation of NF- kβ, JAK-STAT, and JNK-MAPK pathways induced by TNFα, IFN-γ, and aniscomycin, respectively [45]. ORF3 is also known to inhibit lipopolysaccharide (LPS)-induced cytokines and chemotactic factors [8]. In contrast, another study demonstrated ORF3 enhanced IFN production upon poly (I:C) treatment through increased activation of RIG-1, which is suggested to be genotype dependent [46].

Finally, the fourth reading frame (ORF4) has only been described in gt1 HEV and is known to be synthesized solely during endoplasmic reticulum stress [47]. In addition, ectopic expression of this ORF4 from gt1 HEV in the huh7 (human hepatoma) liver cell lines enhanced replication of gt1 (Sar55) and gt3 (P1 and P6) strains [48]. Although it has been reported that ORF4 interacts with RdRp to facilitate HEV replication, further research is required to understand how ORF4 may regulate host immune responses and contributes to enhanced replication.

## 5. Severe Pathogenesis of Hepatitis E in Certain Populations

**Figure 4:**
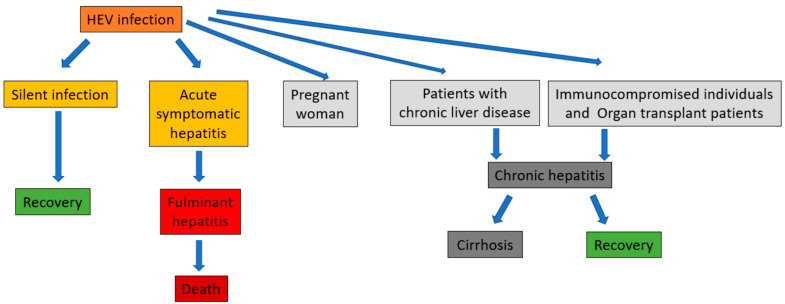
Different disease scenarios seen with HEV.

Pathogenesis of HEV differs between general, pregnant, and immunocompromised individuals (Figure 4). 

Although mainly self-limiting to immunocompetent individuals, HEV is known to be lethal in pregnant women, particularly during the third trimester of pregnancy and in solid organ transplant (SOT) patients prescribed immunosuppressive drugs. 

### 5.1. General Population

Definite reasons behind HEV causing serious disease or producing fulminant hepatitis are still debatable. Host and viral factors are known to play major roles while producing HEV-related disease. 

Liver failure has been related to active stimulation of both Th1 and Th2 immune responses. Higher seroconversion demonstrating anti-HEV IgM and IgG than those of self-limiting infections were noted in patients with fulminant hepatic failure (FHF) [49]. Furthermore, peripheral blood mononuclear cells (PBMCs) from patients with FHF produce higher IFN-γ, TNF-α, IL-2, and IL-10 concentrations after stimulation with ORF2 peptides than do PBMCs from healthy controls [49]. Contrastingly, minimal antiviral cellular response and heightened humoral antiviral responses in patients with fulminant hepatitis E were reported than in patients with uncomplicated infection and control patients [50]. Severe HEV disease was related to a heightened humoral response in both studies. Interestingly, CD4+ T cells were more frequent in the livers of patients with FHF due to HEV and CD8+ T cells have been shown to infiltrate the livers of patients with fulminant hepatitis E. Hence, it is suggested that cytotoxic T cells (CD8+) could be playing a major role in the pathogenesis of fulminant hepatitis. 

Viral factors are considered important in the pathogenesis of HEV as genotype-based differences are seen in infected patients. The severity of an infection could be linked to genotype and/or the sub genotype sequence variations which has been attributed to specific mutations in the ORF1 polyprotein. For instance, HEV gt1 strains from six Indian patients with FHF contained six amino acid mutations in the ORF1 polyprotein (F179S, A317T, T735I, L1110F, V1120I, and FG1439Y) which were not seen in the strains from patients with uncomplicated acute hepatitis E [51]. Similarly, two mutations (C1483W and N1530T) in the HEV gt1 polymerases were found in all 25 patients with acute liver failure but in none of the patients with acute hepatitis E [52]. In addition, three amino acid mutations (V27A, D29N, H105R) in the HEV gt1 methyltransferases were found in 16 patients with acute liver failure, but not detected in uncomplicated acute hepatitis E patients [53]. While these studies lacked a robust sample size, they suggest further surveillance of clinical samples and testing of these sub genotypic mutations in animal model systems are warranted. 

Furthermore, to detail the immune response during HEV infection, the gene expression profile of liver tissues infected with HEV was studied in human liver chimeric mice (uPA-SCID) [54]. These humanized mice are a great tool because they lack a functional adaptive immune system. CXCL9 and CXCL10 are two chemokines involved in leucocyte stimulation and trafficking, and adhesion molecules expression. Their expression was demonstrated to be increased by 24.3-fold and 8.7-fold, respectively. A large number of ISGs (IFI27, IFI44L, IFIT1, IFIT2, IFIT3, ISG20, OAS2, OASL, RSAD2, TAP1, and TRIM22) were directly upregulated upon infection. A twofold to threefold increase of antigen presentation genes (HLA-A, HLA-B, HLA-F, and HLA-J) was observed. Furthermore, the IFI6 gene, which plays a crucial role in the regulation of apoptosis, remained completely unaffected in mice. Despite the robust activation of the innate immune response, the viral infection was not spontaneously cleared [54]. In contrast, another study demonstrated that HEV gt1 and gt3 infections did not elicit innate immune responses and were demonstrated to be highly sensitive to pegylated interferon-α (pegIFNα) in immunocompromised humanized mice [55]. In brief, ISGs’ induction was not observed in untreated HEV gt3 and gt1. However, human-specific ISG transcript levels in mouse liver increased significantly after pegIFNα treatment and induced high circulating human CXCL10 in mouse serum [55]. In addition, animal models possessing both a human liver and human immune system are in ongoing development, but still suffer suboptimal crosstalk between the liver and immune compartment [56]. 

Moreover, risk factors associated with fatal fulminant hepatitis in the general population includes age > 60 years, occupation exposure as seen in swine workers, rural areas with poor water supplies, alcohol consumption, and consumption of pork products (bacon, cured pork meats, and pig’s liver). Furthermore, veterinarians could be at risk for the development of chronic HEV infection [57,58,59]. Similarly, hematological malignancies, age, and a history of pre-existing liver diseases are considered as risk factors for the development of FHF caused by HEV gt1 [60,61]. In addition, HEV gt3 is linked to acute nontravel-associated hepatitis E, which can appear as fulminant hepatitis with encephalopathy and coagulation disorders [62].

### 5.2. In Pregnant Population

Elevated pregnancy mortality has been linked with HEV gt1 and is particularly seen in developing countries [63]. HEV infection with gt1 during the third trimester can lead to maternal mortality in up to 15% to 25% of cases [64]. Recent studies have shown the existence of ORF4 in gt1 HEV and have been hypothesized as a causative factor leading to fatal pregnancy and fetal outcome [47]. This is supported by HEV gt3 being found in pregnant women without lethal pregnancy outcomes, perhaps pertaining to the lack of ORF4, although additional differential factors have not been ruled out. Although the mechanism of liver injury is not clear, it is possible that interplay of hormonal and immunologic changes during pregnancy, along with a high viral load of HEV, renders the woman more vulnerable [65]. Host factors such as immune status, hormone levels, nutritional imbalances, and viral factors have been hypothesized as contributing factors to the poor pregnancy outcome of HEV infection while pregnant.

Pregnancy leads to the changes in the immune system that are designed to protect the embryo and later fetus against the robust maternal immune system. Immunologic changes during pregnancy promote the maintenance of the fetus in the maternal environment by suppression of T cell-mediated immunity, rendering pregnant women more susceptible to viral infections such as HEV [66]. For instance, macrophage activation is known to be suppressed by shifting the Th1-dominated immune response to a Th-2 dominated response (called Th2 bias) to protect the fetus [67]. Th2 bias has been demonstrated in pregnant women infected with HEV; however, its consequences or contribution in the mechanism producing pregnancy pathology are unknown. Furthermore, reduced expression of Toll-like receptor (TLR) 3/TLR7/TLR9 was seen in women with acute liver failure. In addition, phagocytic macrophages were found to be weaker than those of women with acute viral hepatitis E [68]. Nevertheless, the two comparative groups did not have any difference in the phagocytic capacities of monocytes. 

For many decades pregnancy-related hormones have been hypothesized to play a leading role in poor pregnancy outcome when infected with HEV. During pregnancy, levels of progesterone, estrogen, and human chorionic gonadotropin increase as pregnancy advances. HEV-positive pregnant women who develop FHF have higher concentrations of estrogen, progesterone, and β-human chorionic gonadotropin (β-HCG) than HEV-negative pregnant women with FHF or healthy controls [69]. In addition, serum from pregnant women, especially those in the third trimester, enhanced HEV replication by inhibiting estrogen receptors and the synthesis of type ׀ IFNs [70]. Furthermore, high estrogen levels during pregnancy are associated with high HEV titers [71]. In vitro studies suggest that estradiol analogs (17β-estradiol and diethylstilbestrol, DES) facilitate HEV replication in vitro, whereas estrogen antagonist (Tamoxifen) suppresses HEV replication [71]. In addition, HEV infection is known to regulate estrogen signaling pathways by inhibiting the cAMP-PKA-CREB and PI3-AKT-mTOR signaling pathways but is independent of the Ras-Raf-MEK-ERK signaling pathway [72]. Protein–protein interaction studies demonstrate that the helicase of HEV interacts with the estrogen receptor (ERα) to inhibit ERα expression [70,71]. Preterm labor could be attributed to the decline in progesterone [73]. It is suggested that during pregnancy, impaired innate immune responses, reduced progesterone levels, and shifts in immune states may aggravate HEV infection and could result in adverse pregnancy outcomes [74]. Similarly, mutation in the progesterone receptor (PROGINS, progesterone receptor G insert) is known to predispose HEV infection in HIV-positive patients [75]. In contrast, another study reported that mutations in the progesterone receptor (PROGINS) may reduce the symptoms of acute hepatitis E and protect against infection in HIV-infected patients, particularly women [76]. Progesterone-mediated replication enhancement is seen in Huh7-S10-3 cells. The modulation is potentially mediated through SH3-domain containing proteins such as PGRMC1/2, but not likely through immunomodulation of the HEV-induced IFN response studied in vitro [77].

Nutritional status has been observed as one of the major factors contributing to pregnancy-related deaths. Similarly, micronutrients and folate deficiencies coupled with differences in the major histocompatibility complex have been proposed to influence the immune response of pregnant women to HEV. Therefore, HEV infections are benign in pregnant women in Egypt, although they are caused by HEV gt1 [78]. In general, poor maternal nutrition status has been related to adverse birth outcomes [79]. There is a very high risk of preterm delivery in pregnant women with HEV infection, with poor neonatal survival rates [80,81]. During an outbreak in Sudan in 2010 to 2011, among 39 pregnant women with HEV infection there were 14 intrauterine deaths and 9 premature deliveries [82]. In two separate studies from India, 15% to 50% of live-born infants of mothers with HEV infection died within 1 week of birth [80,83]. In early days of childbirth, breast feeding is strongly recommended to support child health and development [84,85]. However, breastfeeding is considered unsafe if the mother has acute hepatic disease or an increased viral load. Hence, there always exists a possibility of transmission from infected breast milk or lesions on the nipple through suckling [86,87]. However, breastfeeding is considered safe in asymptomatic women infected with HEV, despite the presence of anti-HEV antibodies and HEV RNA in the colostrum [87].

**Figure 5:**
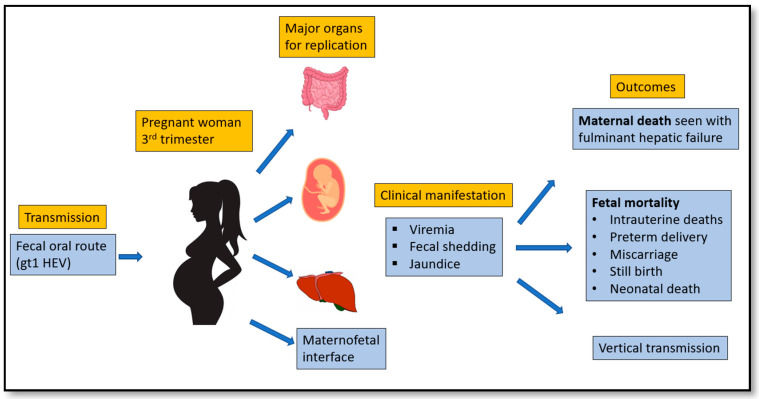
Transmission route, replication organs, clinical manifestations, and outcomes of HEV in pregnant woman.

Viral factors such as HEV RNA concentration have been related to poor pregnancy outcomes. While limited studies have found high HEV RNA concentrations in HEV-infected pregnant women with poor outcomes, there have been other reports demonstrating that only 1 out of 14 pregnant women had detectable HEV RNA [88,89]. Hence, further data is required that would relate the HEV RNA level with the pregnancy pathology. In the last decade, several studies have reported enhanced replication of HEV that could explain higher viral loads in pregnant women than in non-pregnant women [89,90]. Replication in intestinal cells, placental cells (JEG-3), maternofetal interface, liver cells, primary human-derived monocytes, and macrophages in vitro could explain the severity of the HEV while pregnant [5,91,92,93,94]. Summarization of the proposed theory has been shown in Figure 5.

### 5.3. In Immunocompromised Population

The majority of HEV infections in immunocompromised individuals, such as solid organ transplant (SOT) recipients and patients with HIV infection, lymphoma, or leukemia, are likely to progress to chronicity [95]. Immunocompromised individuals, particularly SOT recipients, have been shown to have a higher incidence of HEV, ranging from 0.9% to 3.5%, based on the detection of HEV RNA [96]. However, in 60% of the cases, acute infections turn to chronic in immunocompromised individuals [96]. 

IFN response is not very favorable in spontaneous HEV clearance, as suggested by the inadequate clearance of HEV in response to higher IFN-stimulated genes (ISGs) in renal transplant recipients when compared to ISG response of patients who cleared their HEV [97]. Hence, it can be speculated that increased expression of ISG in patients with a chronic HEV infection favors virus persistence by causing the interferon signaling pathway to be refractory. During HEV persistence, lower concentrations of IL1Rα and soluble IL2R with higher concentration of chemokines is seen [98]. Furthermore, CD2+, CD3+, and CD4+ T cell subsets are significantly lower in chronic immunocompromised patients than in those who spontaneously clear the virus [99]. HIV-infected patients and others with low CD4+ T cell counts are frequently reported to have chronic HEV infections [100,101]. In addition, it is known that gamma delta T cells (γδ T) cells of SOT patients are mobilized during the acute phase of infection and are associated with a favorable outcome in the immunocompromised patients [102]. Because these responses have not been demonstrated in the immunocompetent host, it is suggested that SOT patients mobilize a larger fraction of their immunity due to immunosuppressive therapy. Furthermore, the mobilization and activation of innate cells such as γδ during acute HEV infection in SOT recipients suggest that they could play a role in antiviral response [102]. 

In short, HEV persistence in chronic patients is related to viral factors. Greater quasispecies heterogeneity in ORF1 and ORF2 regions during the acute phase of infection is associated with HEV persistence [98,103]. In comparison to the patients who have cleared HEV spontaneously, K_a_ (rate of non-synonymous substitutions)/K_s_ (rate of synonymous substitutions) ratio, an indirect indicator of the selection pressure on quasispecies, in the M domain of the virus capsid protein, is lower in chronic HEV patients [98]. Similarly, the M domain contains T cell epitopes, highlighting the importance of the cellular immune response for HEV clearance. Additionally, the K_a_/K_s_ ratio of the virus domains containing B cell epitopes in the two groups of patients were not different [103]. 

Cirrhosis is seen in nearly 10% of SOT patients with HEV infection within 3–5 years. Furthermore, chronically infected patients have been found to harbor recombinant HEV-host variants [104,105]. The hypervariable region (HVR) or polyproline region (PPR), regions of these recombinant variants include fragments of human genes of varying origin inter alpha trypsin inhibitor (ITI-H2), ribosomal genes S17 or S19 and tyrosine aminotransferase (TAT). All the variants harboring the S17, S19, or ITI fragment had a replicative advantage in vitro, while the impact of TAT was not studied. Duplications and insertions were also described in the HEV genome [105,106].

Immunosuppressive regimens have been associated with the development of chronic HEV infection. It has been demonstrated that HEV gt3-infected pigs when given cyclosporin, azathioprine, and prednisolone developed chronic HEV infections [107]. In SOT humans, tacrolimus (impairs the specific T cell response) rather than cyclosporin is related to HEV persistence [96]. Furthermore, in vitro data report the promotion of HEV replication by tacrolimus and cyclosporin via inhibiting cyclophilins A and B [108]. Other drugs such as rapamycin and everolimus also promote HEV replication in vitro via inhibition of the mechanistic target of rapamycin (mTOR) demonstrating the PI3K-PKB-mTOR pathway acts as a cell restriction factor [109]. Thus, higher HEV RNA concentrations can be seen in the blood when mTOR inhibitors are given [110].

## 6. In Vitro Models Attempted to Study the Replication and Pathogenesis of HEV

In vitro models are useful tools to study molecular pathogenesis of the pathogen. However, a historical lack of robust replication in cell culture has slowed down the study of HEV molecular pathogenesis. In the last decade, several advances in the cell culture systems have significantly enhanced the replication of HEV in vitro. The advances in HEV cell culture systems include the selection of specific cell line subclones, use of organ derived primary cells, stem cell-based models, and the generation of polarized cell models [6,111,112,113]. Different cell lines used to enhance the replication and the pathogenesis of HEV are mentioned below (Table 1).

## 7. In Vivo Models Attempted to Study the Replication and Pathogenesis of HEV

To understand HEV pathogenesis in humans, several animal models were studied to recapitulate the clinical signs and the tissue specific pathology. In vivo animal models are useful tools for elucidating HEV infection, extrahepatic HEV pathogenesis, virus–host interaction, and evaluation of potential anti-HEV therapies and vaccines. Although hepatic manifestations have been described in HEV infection, extrahepatic manifestations as neurological disorders, renal disorders, hematological disorders, pancreatic disorder, genital disorder, gastrointestinal disorder, and pregnancy associated disorders are also seen (Table 2).

## 8. Conclusions and Future Perspectives

Pathogenesis of HEV infection involves complex molecules providing a favorable environment to HEV for the replication in pregnant otherwise immunocompromised individuals. Although clinical manifestation is mostly hepatological, various studies have demonstrated the ability of HEV to replicate in extrahepatic tissues. While the receptor of HEV have not been fully defined, multiple sensors of innate immunity have been shown to be active inducing IFN and inflammatory response against HEV. However, HEV have developed strategies to counteract the host innate immune response utilizing the viral proteins. Hence, the interplay between host immunity and the virus determines the clinical outcome of the patient. Future studies should target certain areas that need to be investigated with HEV. First, the exact receptors that recognize HEV and allow entry into the cell should be addressed. Second, the mechanisms behind HEV pathogenesis producing worse outcomes in pregnant women must be considered. Third, the role of HEV leading to chronic hepatitis in immunocompromised patients need to be evaluated. HEV coinfection studies with different comorbidities need to be conducted to be prepared for any unusual medical scenario that could be seen in HEV-infected individuals.

## Figures and Tables

**Table 1:** Different cell systems used to enhance the replication and the pathogenesis of HEV.

Cell Line	HEV Genotype	Reference
**Hepatoma Cell Lines**		
PLC/PRF/5	Unknown	Pilot et al., 1987 [114]
	gt4	Tanaka et al., 2009 [115]
	gt1	Takahashi et al., 2010 [116]
	gt3	Shukla et al., 2011 [104]
HepG2	Unknown	Okamoto et al., 2011 [117]
HepG2C3A	gt3	Shukla et al., 2011 [104]
		Capelli et al., 2019 [6]
Huh7.5	gt3	Shukla et al., 2011 [104]
ORF4 expressing huh7 S10-3	gt3, gt1 Sar55	Yadav et al., 2021 [48]
Polarized HepG2C3A	gt3, gt1	Capelli et al., 2019 [6]
**Non-Hepatoma Cell Lines**		
2BS (Human fetal lung diploid fibroblast)	gt1	Huang et al., 1999 [118]
A549 (Human lung epithelial cells)	gt1	Huang et al., 1999 [118]
		Okamoto et al., 2011 [117]
	gt4	Tanaka et al., 2009 [115]
	gt1	Takahashi et al., 2010 [116]
	gt3	Shukla et al., 2012 [119]
LLC-PK1 (Pig kidney cells)	gt3 Kernow-C1	Shukla et al., 2011 [104]
LLC-PK1A (Pig kidney cells)		
SK-RST (Pig kidney cells)		
MDCK (Dog kidney cells)	gt3 Kernow-C1	Shukla et al., 2011 [104]
CRFK (Cat kidney cells)	gt3 Kernow-C1	Shukla et al., 2011 [104]
LLC-RK1 (Rabbit kidney)	gt3 Kernow-C1	Shukla et al., 2011 [104]
Caco-2 (Colon carcinoma)	gt1 Sar55	Emerson et al., 2004 [120]
JEG-3 (Human placental cells)	gt1 and gt3	Knegendorf et al., 2018 [91]
BeWo (Human placental cells)	gt1 and gt3	Knegendorf et al., 2018 [91]
MO3.13 (Oligodendrocytic cells)	gt3	Drave et al., 2016 [121]
**Ex Vivo Transplants**		
Maternal decidua and fetal placenta	gt1 and gt3	Gouilly et al., 2018 [92]
**Primary Cells**		
Primary human hepatocytes (PHHs)	gt3 and gt4	Oshiro et al., 2014 [93]
Immune competent PHHs	gt3 Kernow-C1 P6	Yin et al., 2017 [94]
Human fetal liver cells	gt3 Kernow-C1 P6	Wu et al., 2018 [122]
Primary mouse neurons	gt3 Kernow-C1 P6	Zhou et al., 2017 [123]
**Stem Cells**	gt3 Kernow-C1 P6	Thi et al., 2020 [112]

**Table 2:** Animal models used to recapitulate the clinical signs and the tissue specific pathology.

Parameters	Species	Reference
**Hepatic Disorders**	Rabbit	Parisi et al., 2019 [124]
	Chicken	Kwon et al., 2012 [125]
**Neurological Disorders**	Mongolian gerbils	Shi et al., 2016 [126]
	Rabbits	Tian et al., 2019 [127]
	BALB/c mice	Zhou et al., 2017 [123]
	Rhesus monkeys	Zhou et al., 2017 [123]
**Renal Disorders**	Pigs	Williams et al., 2001 [128]
	NHPs	Geng et al., 2016 [129]
		Huang et al., 2016 [130]
	Mongolian gerbils	Hong et al., 2015 [131]
		Soomro et al., 2016 [132]
	Rabbits	Han et al., 2014 [133]
**Hematological Disorders**	Pigs	Williams et al., 2001 [128]
		Jung et al., 2020 [134]
	Rabbits	Wu et al., 2017 [135]
	Cynomolgus monkeys	Bottino et al., 2018 [136]
**Pancreatic Disorder**	Miniature pigs	Jung et al., 2020 [134]
**Genital Disorder**	Mongolian gerbils	Soomro et al., 2017 [137]
	Rhesus monkeys	Huang et al., 2018 [138]
	BALB/c mice	Situ et al., 2020 [139]
	Rabbits	An et al., 2018 [140]
**Gastrointestinal Tract Disorder**	Pigs	Williams et al., 2001 [128]
	Rabbits	Han et al., 2014 [133]
		Mao et al., 2014 [141]
	BALB/c nude mice	Huang et al., 2009 [142]
**Pregnancy Disorder**	Rabbits	Xia et al., 2015 [143]
	BALB/c mice	Yang et al., 2019 [144]
	Rhesus monkeys	Tsarev et al., 1995 [145]
		Yu et al., 2020 [74]

BALB/c (Bagg albino); NHPs (Nonhuman primates).
